# Isolation and characterisation of alveolar type II pneumocytes from adult bovine lung

**DOI:** 10.1038/s41598-018-30234-x

**Published:** 2018-08-09

**Authors:** Diane Frances Lee, Francisco Javier Salguero, Duncan Grainger, Robert James Francis, Kirsty MacLellan-Gibson, Mark Andrew Chambers

**Affiliations:** 1 School of Veterinary Medicine, University of Surrey, Daphne Jackson Road, Guildford, GU2 7AL England; 2 National Institute of Biological Standards and Control, Blanche Lane, South Mimms, Potters Bar, UK EN6 3QG England

## Abstract

Alveolar type II (ATII) cells play a key role as part of the distal lung epithelium, including roles in the innate immune response and as self-renewing progenitors to replace alveolar type I (ATI) cells during regeneration of the alveolar epithelium. Their secretion of surfactant protein helps to maintain homeostasis in the distal lung and exert protective, antimicrobial properties. Despite the cell’s crucial roles, they remain difficult to study, in part due to inefficient and expensive isolation methods, a propensity to differentiate into alveolar type I cells in culture and susceptibility to fibroblast overgrowth from primary isolations. Published methods of isolation often require specialist technology, negatively impacting the development of *in vitro* models of disease, including bovine tuberculosis (BTB), a serious re-emerging disease in both animals and humans worldwide. We present here a simple and cost-effective method that may be utilised in the generation of bovine primary ATII cells. These exhibit an ATII phenotype in 2D and 3D culture in our studies and are conducive to further study of the role of ATII cells in bovine respiratory diseases.

## Introduction

The alveoli are specialised regions of the distal lung, with a morphology conducive to efficient gaseous exchange. Two types of epithelial cell line the alveolus. Alveolar type I (ATI) cells exhibit a broad, flattened morphology and cover around 95% of the surface area, whilst the cuboidal alveolar type II cells (ATII cells) line the remainder of the alveolus^1^. ATI cells provide a gas exchange interface with the underlying endothelium, whereas ATII cells serve as both progenitors of ATI cells and also play a critical role in maintaining the homeostasis of the alveolus^2^. The latter role is fulfilled by the secretion of surfactant proteins from specialised organelles within ATII cells, so-called ‘lamellar bodies’, into the alveolar space. This maintains surface tension and prevents alveolar collapse, whilst contributing to the varied functions of the ATII cells^3^. These include the innate immune response, during which surfactant proteins A and D have been shown to bind bacteria, viruses and fungi and facilitate their removal by alveolar macrophages^4,5^, a function that led to the coining of the term ‘collectins’. The ATII cell is the only epithelial cell of the lung which synthesises and releases all four surfactant proteins A, B, C and D, with surfactant protein C being unique to the ATII cell^6^. This unique marker of ATII cells has recently been shown to attenuate the Janus tyrosine Kinase (JAK) - Signal Transducer and Activator of Transcription (STAT) inflammatory pathway^7^, providing further key insights into a process previously shown to be associated with the classical activation of macrophages^8^.

Studies utilising ATII cells, or cell lines derived from them, are vital to the elucidation of host-pathogen interactions, including diseases falling under the One Health remit, such as tuberculosis, in the top ten causes of death worldwide^9^. The causative agent of bovine tuberculosis (BTB), *Mycobacterium bovis*, can cause progressive disease in most warm-blooded mammals, including humans, with the WHO estimating that 3.1% of all human cases of tuberculosis are caused by BTB^9^. Persistence of the disease in cattle has been attributed to, amongst other factors, wildlife reservoirs of the infection and the limitations of current diagnostic tests^10^. To overcome these challenges, new *in vitro* models of the alveolus are required to enable comparative studies between species and evaluate the role of the ATII cell in the initial stages of BTB pathogenesis.

Evaluating the role of the ATII cell in bovine pulmonary diseases such as BTB requires an efficient method of ATII cell isolation - a process often fraught with challenges. These include the impurity of primary isolations, variable levels of cell viability, and fibroblast outgrowth in studies that require longer culture periods. Current models of the human distal lung commonly utilise cell lines, including the human lung carcinoma cell line A549; however, these have been shown to have an unstable phenotype and recent studies offer conflicting evidence as to their suitability as an *in vitro* model^11^. Furthermore, it can be argued that bovine ATII cells carry more relevance to bovine respiratory disease, particularly given the anatomical differences previously observed between mammals^12^.

We present here a simple isolation technique that requires no specialist equipment (as with flow cytometry, or magnetic separation), facilitating transfer of the procedure to other laboratories. Furthermore, our procedure is applicable to other species with the potential to contribute to a reduction in the number of studies requiring the use of live animals.

## Results

### Isolation of ATII cells

**Figure 1:**
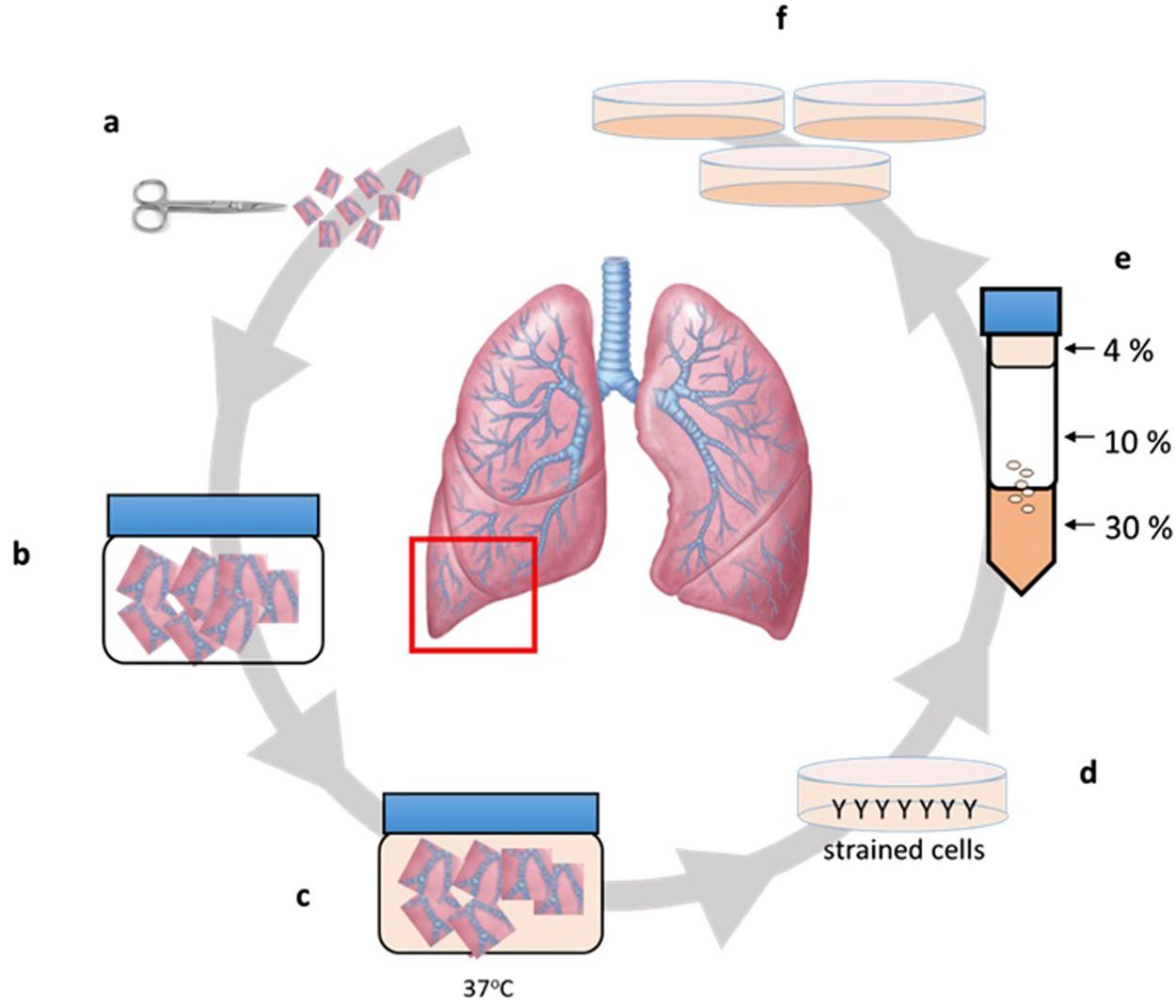
Schematic of the purification procedure from animal to culture. (**a**) Lungs were obtained directly from slaughter and taken back to the laboratory, where aseptic dissection took place. (**b**) The tissue was then washed repeatedly with DPBS containing EDTA and penicillin/streptomycin. (**c**) Tissue was digested at 37 °C, then filtered sequentially through mesh of decreasing pore sizes. (**d**) The filtrate was overlaid onto bovine IgG-coated bacterial grade petri dishes to remove fibroblasts and macrophages. (**e**) Non-adherent cells were then loaded in 4% Percoll™ onto a Percoll™ gradient, overlaying onto 10–30% Percoll, which generated an ATII enriched emulsion at the 10–30% interface. (**f**) These cells were then washed, counted and plated out in SAGM, containing 100 U/mL penicillin/streptomycin.

**Figure 2:**
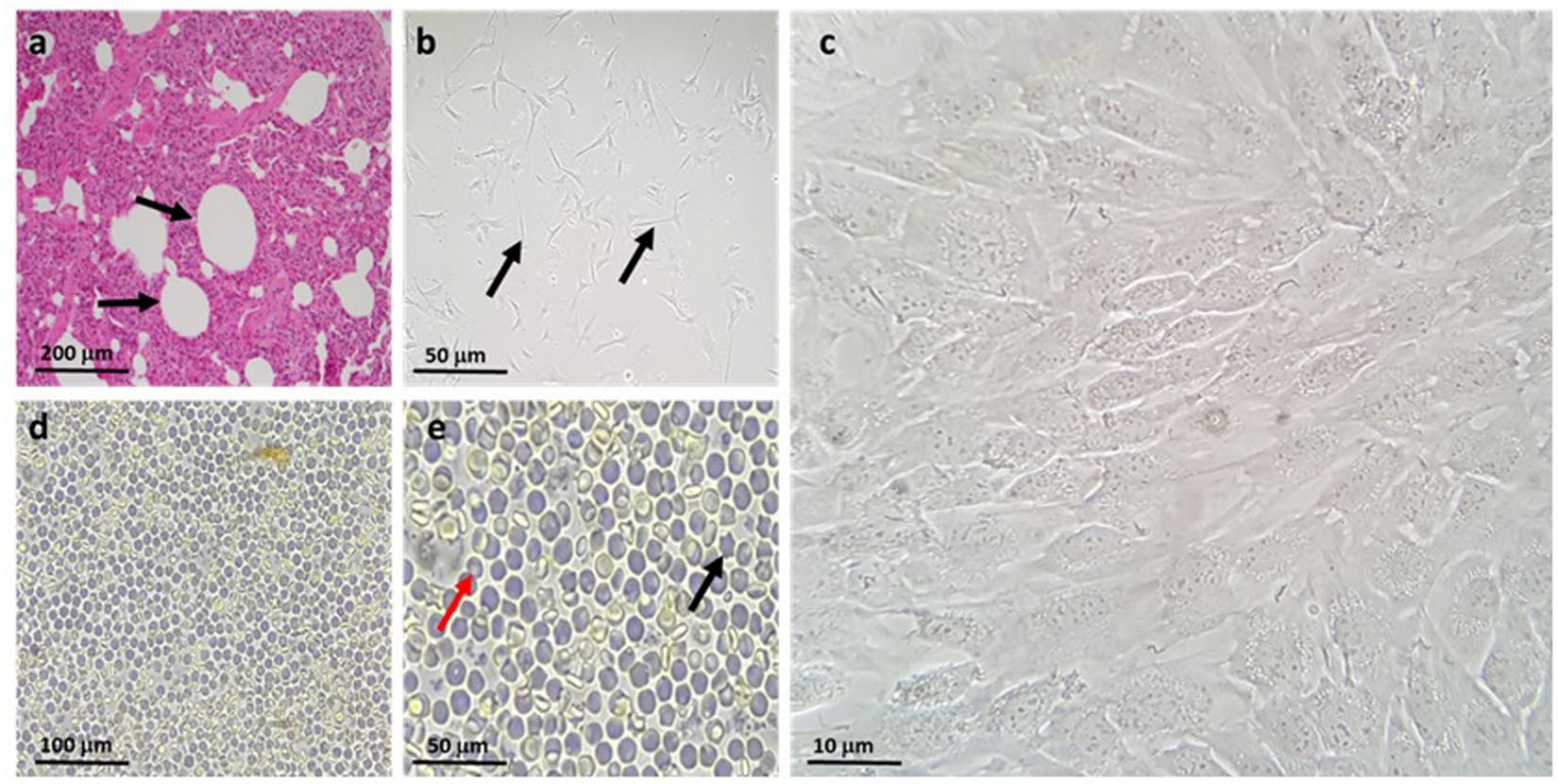
Isolation was performed from the distal lobe of the right lung. (**a**) H&E staining (see methods) was used to verify the health status and phenotype of the region dissected. Multiple alveolar ducts are marked by arrows. (**b**) The enriched fraction obtained by Percoll gradient, with some residual fibroblast contamination (arrows). (**c**) Representative image of the cobblestone morphology observed in isolated ATII cells. (**d**) Cells isolated in the pellet fraction of the Percoll gradient, including macrophages and erythrocytes, with negligible epithelial content. (**e**) Percoll fraction image acquired using a 40 x objective, highlighting a macrophage (black arrow) and convex erythrocyte (red arrow). Images representative of three independent isolations.

**Figure 3:**
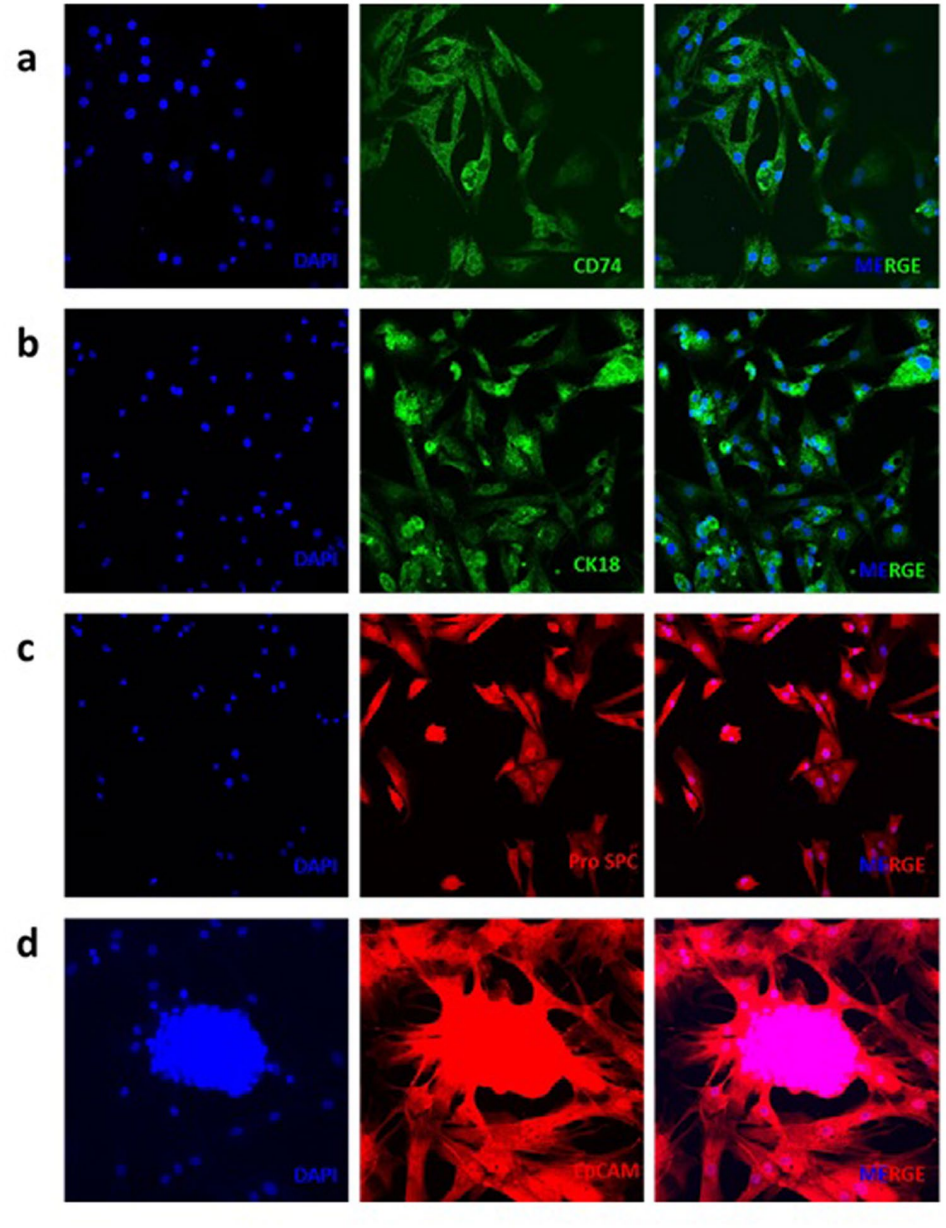
IF analysis of isolated cells cultured on 8-chamber slides over a period of 48 hours. (**a**) The cell surface marker CD74, (**b**) cytoplasmic cytokeratin 18 (CK18) and (**c**) surfactant protein SPC are specific to the ATII phenotype, whilst epithelial cell adhesion molecule (EpCAM) (**d**) is a general identifier of the epithelial cell phenotype. Images representative of three independent isolations.

We isolated cells from the right distal lobe region of adult bovine lungs (Fig. 1). Haematoxylin and Eosin (H&E) staining confirmed the presence of alveolar ducts (Fig. 2a, arrows) in freshly dissected tissue and that the lungs were free of apparent pathologies. Isolation was achieved using a relatively simple method of enzymatic digestion of dissected lung tissue, each time taking from the right distal lung to ensure consistency and direct comparison during method optimisation. This was followed by selective adherence (IgG ‘panning’) to remove most macrophages and contaminating fibroblasts, a method first published by Wysocki *et al*. for the purpose of isolating Fc-receptor expressing lymphocytes^13^. By loading IgG ‘panned’ cell suspensions onto a Percoll™ gradient, a distinctive band of cells was achieved at the 10–30% Percoll™ interface, in accordance with the results reported for human ATII cells by Mao *et al*.^14^ Yield was determined to be 5.63 × 10^6^ ± 0.87 cells total; an average taken from three separate isolations. Viability was assessed using the trypan blue exclusion method and was found to be 53 ± 9%. The enriched fraction contained an 85–90% pure epithelial-like phenotype (Fig. 2b), with an approximated 10–15% fibroblast (spindle-like, light-refractive morphology, arrows) and minor macrophage contamination. Epithelial-like cells formed colonies which were characterised by the cuboidal ‘cobblestone’ cell morphology apparent under light microscopy (Fig. 2c). A culture of the pellet fraction obtained following Percoll™ gradient centrifugation showed a mixed population with negligible epithelial cell content, predominantly containing macrophages (Fig. 2d,e, black arrow) and erythrocytes (red arrow) at day one post-isolation. Immunofluorescence analysis of the enriched cells cultured for 48 hours on 8-chamber slides demonstrated expression of the commonly used ATII markers cluster of differentiation 74 (CD74) (Fig. 3a), cytokeratin 18 (CK18) (Fig. 3b), pro-surfactant protein C (Pro SPC) (Fig. 3c), and epithelial cell adhesion molecule (EpCAM) (Fig. 3d).

### Permeable membrane and 3D Cell culture

**Figure 4:**
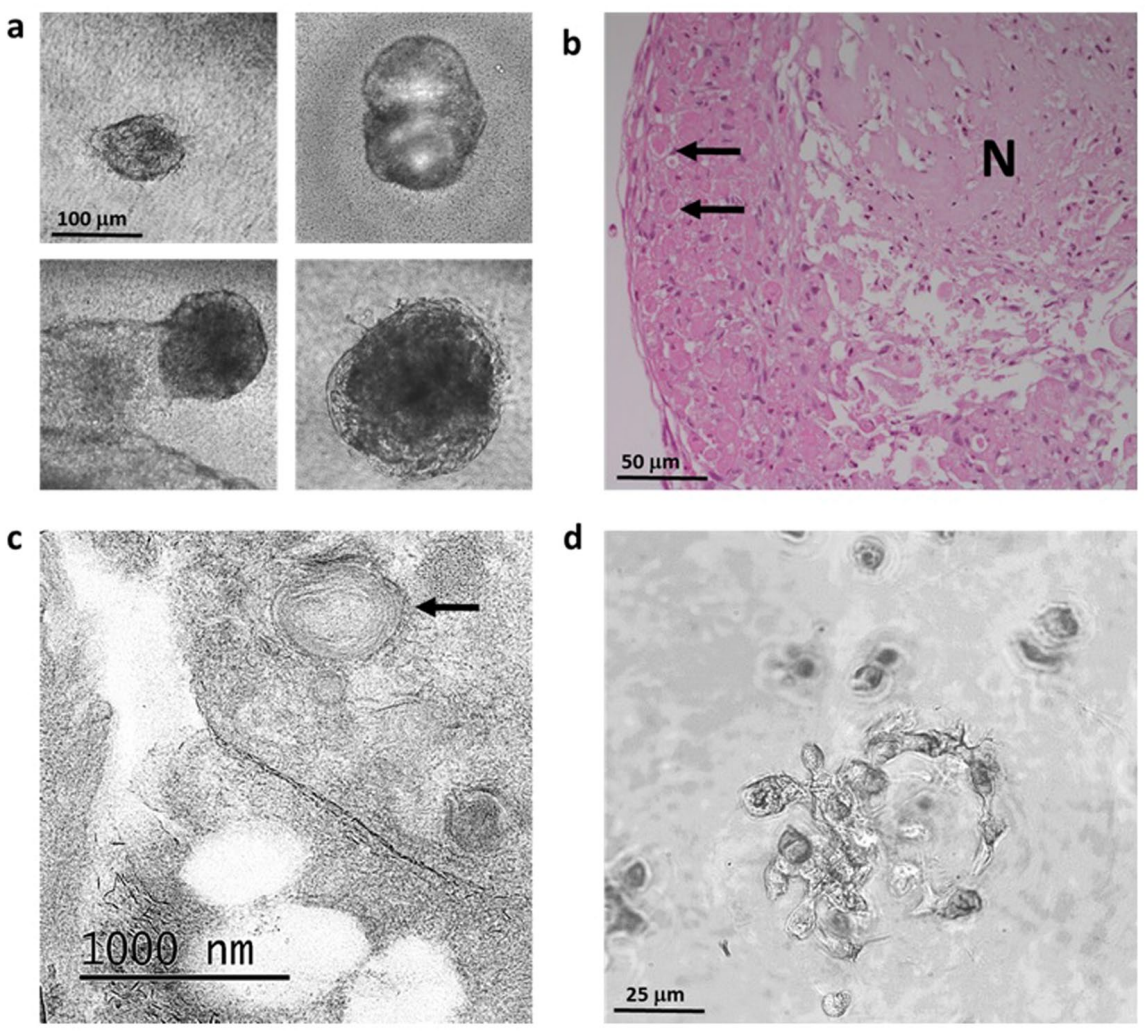
Three dimensional (3D) culture of isolated cells on permeable inserts and embedded in Matrigel. (**a**) Isolated ATII cells were seeded onto Matrigel-coated (1:10 dilution) Transwell™ 12 mm inserts and cultured for 2 days submerged, followed by 8 days at air-liquid interface. (**b**) H&E staining of two week old organoid cross-sections containing necrotic (N) material, with live peripheral cells staining strongly for large spherical vesicles (arrows), consistent with those containing surfactant^17^. (**c**) Representative TEM image, as carried out on cross sections of cells cultured on 6 mm Greiner Thincerts, confirming presence of lamellar bodies. (**d**) Ringed structures surrounding the ‘lumen’ in isolated cells cultured as a 3D suspension. Images representative of three independent isolations.

It has been demonstrated previously that ATII cells form alveolar-like structures when cultured in a 3-dimensional format, such as Matrigel™^15^. To generate further evidence that the isolated cells exhibited an ATII phenotype, they were seeded onto 12 mm permeable supports (both Corning Transwell™ and Greiner Thincert™) coated with a 1:10 dilution of Matrigel™ in small airway growth medium (SAGM). Following an initial period of 48 h in submerged culture, medium was removed from the apical chambers and the cells cultured for a further eight days. Removal of media from the apical chamber did not generate long term air-liquid interface (ALI) and media seepage was observed through both membranes within 15 minutes of removal from the apical chamber. Nevertheless, cells formed three-dimensional organoids on both supports during the culture period (Fig. 4a). These organoid structures were reminiscent of those reported by Lee *et al*.^15^ and Barkauskas *et al*.^16^ for murine and human ATII cells, respectively. Organoids left to grow for longer periods (2 weeks) were fixed, sectioned and subjected to H&E staining. These were found to contain necrotic material in the centre of the ‘lumen’, with signs of proliferating cells localised to the periphery of the structure (Fig. 4b). Some of these proliferating cells contained large, strongly-stained vesicles; a staining characteristic of ATII cells^17^. Sectioning and transmission electron microscopy (TEM) analysis of two week cultures revealed striated lamellar bodies, the hallmark identifier of ATII cells (Fig. 4c). To explore how these ATII cells behaved when embedded in an extracellular matrix (ECM), they were seeded in 24 well plates, suspended in Matrigel™ (50 μL) overlaid with small airway growth medium (SAGM) (500 μL), as used in other organoid culture models^18^. Culturing this way provided a true three-dimensional architecture in which the cells were observed to form ring like structures, with the beginnings of a lumen in the centre (Fig. 4d). Collectively, these analyses provided substantial evidence that our cultures contained ATII cells that retained their true phenotype.

### Analysis of Gene Expression

**Figure 5:**
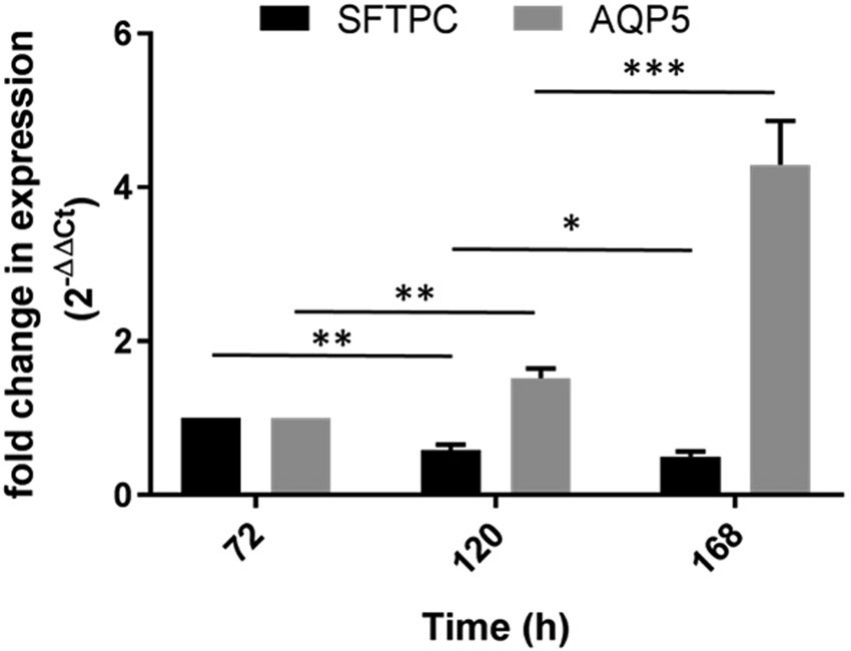
Comparative analysis of gene expression, comparing *SFTPC* (ATII marker) with that of *AQP5* (ATI marker). Expression of each gene was normalised to the housekeeping control, *GAPDH*. Statistical analysis found a significant reduction in *SFTPC* between 72 and 120 h, with further reduction observed at 168 h. An inverse relationship was seen between *SFTPC* and *AQP5* over time, with a concurrent increase in *AQP5* at the same time points. Mean ± SD, n = 6 (across two experiments). *P ≤ 0.05; **P ≤ 0.001; ***P ≤ 0.0001 (two tailed *t* test, unpaired).

It has been previously reported that the expression of surfactant protein C (*SFTPC*) decreases over time in submerged or 2D culture of ATII cells on plastic^14,19,20^, along with other ATII markers. During the current study, an ALI of ATII monocultures was not achieved and therefore ATII cultures seeded on membranes were submerged at each time point. To monitor the expression of *SFTPC* in a submerged membrane culture of ATII cells and to study differentiation of ATII into ATI, we determined *SFTPC* gene expression from mRNA isolates at different time points, in conjunction with the ATI marker aquaporin 5 (*AQP5*)^21^ and normalising to glyceraldehyde 3-phosphate dehydrogenase (*GAPDH*) as an internal housekeeping gene control. Cells were harvested from inserts at 72, 96 and 168 h and processed as outlined in the methods section. *SFTPC* gene expression was shown to be significantly down-regulated, with a 0.6 fold change in expression at 120 h (unpaired, two tailed *t* test, P ≤ 0.01), relative to 72 h (Fig. 5); a further reduction in *SFTPC* was observed at 168 h, with a 0.5 fold change in expression relative to 72 h (P ≤ 0.05). An inverse relationship to *SFTPC* expression was observed for the ATI marker *AQP5*, which was significantly upregulated 1.5 fold from 72 to 120 h (P ≤ 0.01), increasing further at 168 h to a 4.3 fold change in expression relative to that of 72 h (P ≤ 0.01). The inverse and dynamic relationship between SFTPC and AQP5 points to differentiation of ATII to ATI, a key feature of the role of ATII cells as progenitors of ATI^22,23^.

### Fibroblast removal

**Figure 6:**
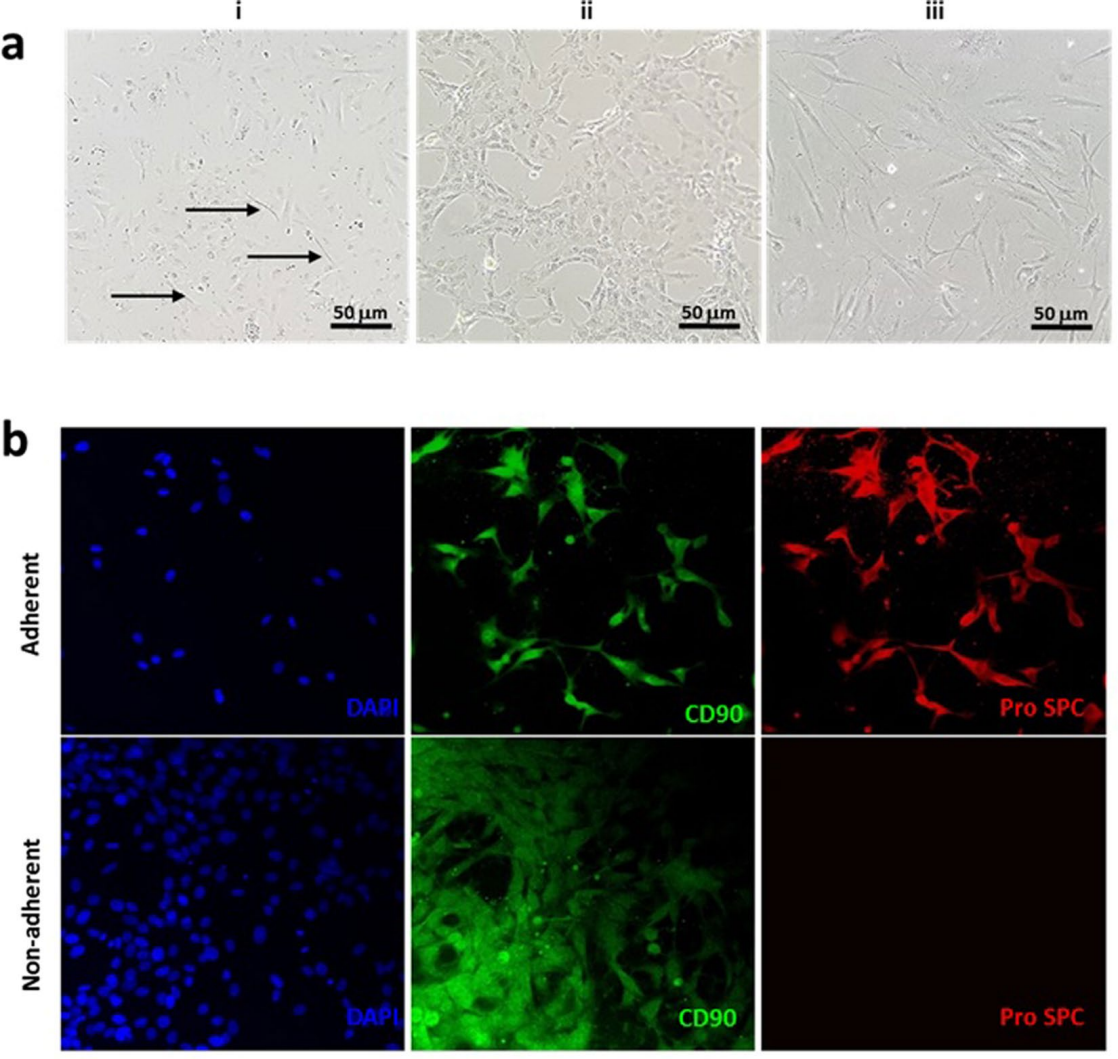
Removal of contaminating fibroblasts from cultures of isolated ATII epithelial cells. (**a**) (i) Fibroblasts, residual from the isolation process, are highlighted by arrows. Cells subjected to HBSS treatment for a minimum of 30 minutes, result in an adherent and non-adherent phenotype. (ii) Cobblestone morphology exhibited by adherent cells, indicative of epithelial cells. (iii) Non-adherent cells with a spindle-like morphology, indicative of fibroblast enrichment. (**b**) IF staining with the ATII marker Pro SP-C and co-stain with CD90, a surface marker previously identified in both epithelial^1^ and fibroblast^41^ cultures; adherent cells are positive for both markers, whilst non-adherent cells appear negative for Pro SP-C expression. Images representative of three treatments, each of 30 minutes duration.

Fibroblast contamination and outgrowth has been repeatedly reported to present difficulties in the maintenance of uniform primary cultures of isolated epithelial cells^24,25^. In the current study, passaged cultures of ATII cells were observed to contain a number of fibroblasts (Fig. 6a), potentially an issue in downstream applications, such as ALI cultures or when monitoring the phenotype or genotype of isolated cells in homeostasis or disease processes. We therefore further enhanced the ATII isolation method by introducing a downstream step to remove fibroblast outgrowth, using Hanks’ balanced salt solution (HBSS) incubation^25^ and cold trypsinisation^26^. HBSS was found to effectively remove fibroblasts within half an hour of treatment, as assessed by cell morphology (light microscopy) and expression of markers by immunofluorescence (Fig. 6b). Cells which remained adherent following HBSS treatment formed colonies that displayed a ‘cobblestone’ appearance characteristic of epithelial cell cultures (Fig. 6a(ii)), whilst cells recovered from the HBSS supernatant displayed a light-refractive spindle morphology (Fig. 6a(iii)). These were aligned as tightly packed ‘fibres’. Cells removed by HBSS incubation stained negatively for the ATII marker pro SP-C, confirming that mostly fibroblasts had been removed. Fibroblast removal in this way allowed the continued use of isolated ATII cells up to their proliferative capacity (the current study has used ATII cells up to passage 11), when cultured under appropriate conditions so as to maintain the type II phenotype. By contrast, cold trypsinisation resulted in the detachment of all cells from the tissue culture surface, therefore this technique was not employed further.

## Discussion

The study of early interactions between infectious agents and ATII cells promises to generate crucial findings relevant to the pathogenesis of infectious respiratory disease, vital for the development of successful disease interventions. However, such studies require reliable *in vitro* models of the alveolus, beginning with isolation of the cells in a consistent and efficient manner. We have developed a cost-effective, simple and reproducible method with which the isolation of ATII cells may be achieved from adult bovine lungs. Our protocol has wide applications, not least the ease of transfer between species and laboratory groups globally.

Whilst isolation methods of ATII cells exist, many are tailored to biopsies, donor tissue or smaller animals, therefore are not conducive to studies involving large animals such as bovines. To determine a suitable method for the isolation of ATII cells from bovine lungs, we first investigated fluorescence activated cell sorting (FACS), such as employed by Gereke *et al*., for the isolation of murine ATII cells^27^. This requires optimisation and validation of the instrument parameters, antibodies with outstanding specificity and affinity and also a relatively close proximity of the instrument to the location of dissection and downstream applications. For the current study, the logistics of lung acquisition, transport to the laboratory, dissection and transport of samples between facilities made FACS a less favourable option. We did perform an isolation using FITC-labelled CD74 as a marker for ATII cells (further details in the Supplementary Information); however, our isolation yielded mostly cell debris (see Supplementary Fig. 1). We also explored the option of magnetic sorting using anti-CD74 FITC, a technique previously utilised successfully in rat ATII isolation^28^. Again, mainly debris was found in the CD74-positive fraction (see Supplementary Fig. 2).These approaches were discounted from further isolation studies as it was discovered that the enzymatic digestion of bovine lung tissue was detrimental to the integrity of the markers chosen, resulting in non-specific binding of cell debris to CD74. Interestingly, Gedye *et al*. discovered during their own studies that both trypsin and collagenase were detrimental to their profiling of surface markers^29^. We included collagenase due to the relatively high collagen content of bovine lungs^30^, whilst trypsin was included as it was reported by Dobbs *et al*. to improve cell yield^3,6,31,32^. Since the availability of suitable cell-specific markers for bovine ATII cells is limited, alternatives to FACS and magnetic sorting were pursued.

The process of lymphocyte removal by IgG^13^ has been utilised successfully as part of the isolation of ATII cells in previous studies^33^. We found that panning was an effective tool for the removal of the majority of lymphocytes and fibroblasts (Fig. 2d), on account of their greater affinity for bacterial-grade plastic^33^. Refinements in future studies might include antigen-directed antibodies directed towards multiple targets^27^.

Fibroblast outgrowth is a common feature of primary cell culture. Traditionally, these are removed by adherence, trypsinisation, or Percoll™ gradients^34,35^. In 1983, Pal *et al*. reported problems with both adherence and trypsinisation, in that the former did not remove all fibroblasts and the latter removed all cell types^25^. The solution of Pal *et al*. was to make use of the fibroblast’s sensitivity to HBSS incubation. In our studies, we determined the optimum treatment time to be 30 min (Fig. 6). We additionally employed the cold trypsin method; however we found that cold trypsinisation removed a substantial amount of epithelial cells. We propose that cold trypsinisation should only be used when cells are isolated in bulk, or where pure cultures are required.

It is commonly reported that ATII cells lose their phenotypic characteristics when cultured over time in 2D, resulting in a flattened ATI-like morphology and phenotype^32,36^. Previous studies have reported that the culture of ovine^37^ and human^36^ ATII cells at ALI resulted in cells retaining their characteristics, including secretion of surfactant proteins and cuboidal shape. To this end, we seeded bovine ATII cells onto Matrigel™-coated polyester inserts and cultured under submerged conditions for 48 h, removing apical medium thereafter. The ATII cells did not achieve ALI, instead forming organoids on the insert surface (Fig. 4a). These were found to contain necrotic material in the ‘lumen’, with evidence of proliferation at the periphery, as shown by H&E staining (Fig. 4b). To assess the behaviour of bovine ATII cells under true 3D format, we seeded ATII cells embedded in Matrigel™ (100%). Cells cultured in this way formed alveolar-like structures as reported previously^14^. The reason our cultures did not achieve ALI is unclear; we postulate that it may be due to interspecies differences, which have been previously shown to affect study outcomes^38,39^, or due to different culture conditions^33^.

## Methods

### Isolation of alveolar type II (ATII) epithelial cells

Lungs were excised from freshly slaughtered cattle less than 24 months of age, at a local abattoir facility under existing licensed slaughter procedures. These were transported directly to the laboratory. The right distal lung was used for all cell isolations, taking a 5 cm^3^ piece for histological confirmation of healthy tissue. Further dissection of the same region of lung was performed within a Class II MBSC, upon approximately 100 g of tissue. Tissue was dissected into pieces no greater than 3 mm^3^. These were placed into 50 mL conical tubes to the 5 mL mark of each tube and filled to 40 mL with wash buffer (Dulbecco’s Phosphate Buffered Saline (DPBS) containing 100 U/mL each of penicillin and streptomycin). Tubes were inverted to wash the tissue and the supernatant discarded, repeating for five washes. Each tube was filled to 40 mL with wash buffer containing 2 mM EDTA and shaken vigorously, again discarding supernatant. This step was repeated twice more before progression to the digestion phase.

Digestion solution (20 mL) (DPBS containing 100 U/mL each of penicillin and streptomycin, 0.008% (w/v) elastase, 0.2% (w/v) collagenase, 0.005% (v/v) DNAse Type I (2000 KU/mL, Sigma D5025) and 0.05% (w/v) trypsin was added to each tube. Digestion was carried out for 30 minutes at 37 °C with rocking. Enzymatic activity was neutralised with an equal volume of Dulbecco’s modified Eagle Medium/Ham’s F12 (DMEM/F12) containing 25% FBS and 0.01% (v/v) DNAse I (2000 KU/mL, Sigma D5025). The resulting suspension was filtered sequentially through 100, 50 and 25 μm filters and the cell suspension collected in 50 mL tubes. Cells were spun at 300 × *g* for 10 minutes at room temperature and resuspended in adhesion solution (1:1 DMEM/F12/Small Airway Growth Medium (SAGM) (PromoCell GmbH, Heidelberg, Germany)), 5% FBS, 0.025% (v/v) DNAse I.

Macrophages were removed by overlaying the cell suspension onto bacterial-grade petri dishes coated overnight at 4 °C with bovine IgG (5 μg/mL) in 0.05 M Tris, pH 9.5, for 1 hour at 37 °C, rocking after 30 minutes to redistribute non-adhered cells. Non-adherent cells were spun at 300 × *g* for 10 minutes and resuspended in 6 mL 4% (v/v) Percoll™, 4% (v/v) FBS, 0.01% DNAse. The cell suspension was overlaid (1 mL per tube) onto a gradient consisting of 6 mL 30% ‘heavy’ Percoll™ and 8 mL 10% ‘light’ Percoll™. Gradients were spun at 400 × *g* for 20 minutes at 4 °C, using the lowest acceleration level and setting the brake to ‘off’. The enriched cell layer at the 10–30% interface was removed and resuspended in DPBS containing 100 U/mL each of penicillin and streptomycin, combining cells in a total volume of 50 mL. Cells were spun at 300 × *g* for 10 minutes at room temperature and resuspended in SAGM. Enriched cells were then plated onto tissue culture treated 6-well plates and cultured at 37 °C, 5% CO_2_, monitoring for viability and morphology and passaging as appropriate until further analysis.

### Haematoxylin and Eosin staining

Dissected distal lung was fixed overnight in 10% neutral buffered formalin, then embedded in paraffin. Sections of 4 μm thickness were cut and subjected to dewaxing and staining as per standard procedure. Briefly, sections were dried and dewaxed in xylene (three soaks of 3 min duration), followed by rehydration (2 min soak in 100% ethanol, two soaks of 2 min each in 95% ethanol and final rinse in distilled water, 1.5 min). Staining was achieved using Mayer’s haematoxylin (5 min), running tap water (2.5 min), 1% acidified alcohol (10 s), running tap water (2.5 min), 95% ethanol (30 s), eosin (2 min) and running tap water (20 s). Samples were dehydrated for mounting (three times 100% ethanol, 30 s followed by xylene 5 min, changing at 3 min). Slides were mounted in DPX mounting medium and a cover-slip overlaid for analysis on a Nikon Eclipse Ci upright microscope. Alveolar-like organoid structures were fixed in 10% neutral buffered formalin for 1 h, embedded in paraffin and processed as per lung tissue.

### Transmission Electron Microscopy

For transmission electron microscopy (TEM), samples were fixed in 4% paraformaldehyde (PFA) for 1 h, washed with DPBS before staining with 1.5% (w/v) osmium tetroxide overnight. Samples were again washed with DPBS, then stained with a 1.5% (w/v) uranyl acetate solution. The insert membranes were then dehydrated in an acetone series and infiltrated using a graded propoxylene/epoxy resin (TAAB, UK) followed by 100% epoxy resin overnight, as per manufacturer’s instructions. Following trimming, inserts were placed into silicone moulds with fresh resin and cured at 65 °C for 48 h. Sectioning was performed using a UC6 ultramicrotome (Leica Microsystems, Milton Keynes, UK). Sections (50 nm) were collected onto formvar-carbon coated gold TEM grids and imaged using a JEOL 2100 transmission electron microscope (JEOL UK Ltd, UK), equipped with a Gatan 4 K ultra scan CCD camera (Gatan, USA).

### Removal of fibroblasts

Hanks’ balanced salt solution (HBSS) was added to cultures suspected of fibroblast overgrowth *in situ* and the plates or flasks incubated at 37 °C for a minimum of 30 minutes and maximum of 1 hour, checking the culture at 15 minute intervals for signs of detachment. Non-adherent cells (fibroblasts) were removed and the adherent cells (enriched ATII) washed with DPBS and fed with fresh SAGM or passaged as appropriate. In subsequent cultures, where fibroblast overgrowth was minimal or undetected, cold trypsin (0.25%) was added to the culture for a maximum of 2 minutes, before removing, washing with DPBS and adding fresh trypsin as per standard passage protocol.

### Immunofluorescence microscopy

Cells seeded onto 8-chamber slides were cultured for 48 hours, to confirm the identity of ATII cells. For immunofluorescence (IF) analysis, cells were washed with DPBS and fixed in 4% PFA for 15 minutes. Cells were rinsed three times with DPBS then permeabilised in 0.1% triton X-100 for 15 minutes. Blocking was performed for 1 hour at room temperature in DPBS/5% normal goat serum/0.1% triton X-100. Primary antibodies were diluted in blocking buffer, applied to cells and incubated overnight at 4 °C. Cells were rinsed three times with DPBS and secondary antibody applied for 1 hour at room temperature in the dark. Cells were again rinsed three times with DPBS and the chamber gasket removed from the slide. Cells were mounted with Prolong® Gold Antifade reagent with 4′,6-diamidino-2-phenylindole (DAPI) (P36941, Thermo Fisher Scientific, Waltham, MA) and imaged using a Nikon Eclipse Ti confocal microscope. Primary antibodies were as follows: pro-surfactant protein C (Pro SPC) (ab3786, Millipore, Burlington, MA; 1:100); cytokeratin 18 (CK18) (sc32329, Santa Cruz, Dallas, TX; 1:100); cluster of differentiation 74 – FITC conjugated (CD74-FITC) (sc47742, Santa Cruz, Dallas, TX; 1:100); epithelial cell adhesion molecule (EpCAM) (orb10618, Biorbyt, Cambridge UK; 1:100); S100 calcium-binding protein A4 (S100A4) (810101, Bio Legend, San Diego, CA; 1:100). Secondary antibodies were Goat anti-Rabbit IgG (H + L), Texas Red conjugated and Goat anti-Mouse IgG (H + L) Secondary Antibody, FITC conjugated (both Thermo Fisher Scientific, Waltham, MA) diluted 1:200. Mouse and rabbit IgG1 isotype controls were used accordingly.

### Membrane and 3D cell cultures

Enriched ATII cells were seeded onto Transwell-Clear™ 12 mm inserts, coated with a 1:10 dilution of Matrigel™ (Corning Incorporated, Corning, NY) in SAGM, at a seeding density of 5 × 10^5^ cells per insert in 500 μL SAGM. Each insert was suspended in a 12-well plate, with 1500 μL SAGM added to the basolateral chamber. At 48 hours, apical medium was removed and the inserts fed from the basolateral aspect only every two days, for a total culture period of ten days. For TEM analysis, cells were seeded onto the apical surface of Greiner 6 mm Thincerts™ at a seeding density of 1 × 10^5^, in 100 μL SAGM. Inserts were suspended in a 24-well plate, with 600 μL SAGM added to the basolateral chamber. Cells were then cultured as for Transwell-Clear™ cultures.

Alternatively, enriched ATII cells were seeded in 50 μL Matrigel™ droplets at a seeding density of 5 × 10^4^ cells per droplet. After polymerisation (approximately fifteen minutes at 37 °C), the droplet was submerged in 500 μL SAGM, culturing for 10 days, exchanging SAGM every 2 days. Cultures of both types were imaged using a Zeiss Axiovert 25 inverted microscope in brightfield mode.

### RNA extraction and quantitative real-time reverse transcription PCR (qRT-PCR)

For relative quantification of cell-specific markers, ATII cells were seeded onto Transwell-Clear™ 12 mm inserts as before and cultured over a time course, harvesting RNA from cells at 72, 120 and 168 hours. At each time point, cells were lysed using TRIzol® reagent (Thermo Fisher Scientific) and total RNA extraction performed using the phenol/chloroform extraction method. Briefly, TRIzol® was incubated with the cells for 5 minutes, followed by scraping of the insert to recover lysate. Fresh TRIzol® was used to make up volume to 1.5 mL in a 2 mL microtube, before adding 0.3 mL chloroform. The tube was shaken vigorously, then stood at room temperature for 2 minutes. Tubes were spun at 12,000 × *g* for 15 minutes at 4 °C and the aqueous phase removed into a fresh 2 mL microtube. Isopropanol (0.5 mL per 1 mL aqueous phase) was added and the tube gently mixed by inversion. RNA was allowed to precipitate for 10 minutes on ice before pelleting at 12,000 × *g* for 10 minutes at 4 °C. The supernatant was aspirated and the pellet washed twice with 75% ethanol (ice cold), with a centrifugation step of 7,500 × *g* for 5 minutes at 4 °C in between, followed by a drying period of 1 hour, before resuspending in 20 μL RNAse free water. Total RNA was quantified using a Biodrop μLITE spectrophotometer and 500 ng used to generate complementary DNA (cDNA) in a reverse transcriptase reaction using the qScript cDNA synthesis kit (Quanta Bio, Beverly, MA), according to the manufacturer’s protocol, on a Techne 3Prime Personal Thermal Cycler (Cole Parmer Instrument Company, London, UK). Quantitative polymerase chain reaction (qPCR) was performed on approximately 60 ng (by RNA quantification) of each sample, using Brilliant III™ Ultra-Fast Mastermix (Agilent Technologies, Santa Clara, CA) according to the manufacturer’s protocol and the following TaqMan™ Gene Expression Assays; SFTPC accession number Bt03259429_g1 fluorescein amidite (FAM) labelled, AQP5 accession number Bt04302387_m1 FAM labelled and GAPDH (housekeeper) accession number Bt03210911_g1 2′-chloro-7′phenyl-1,4-dichloro-6-carboxy-fluorescein (VIC) labelled (Thermo Fisher Scientific), as duplex reactions and in triplicate, using a CFX96 Touch Real-Time PCR Detection System (BioRad, Hercules, CA). Fold change in expression was calculated using the Livak^40^ method (2^−ΔΔCt^).

### Statistics

All statistical analysis was performed using GraphPad Prism version 7.03 for Windows, GraphPad Software, La Jolla California USA, www.graphpad.com. The relative mRNA level in the qRT-PCR analysis was represented by mean ± SD, n = 6 across two experiments performed on different days. The significance of a fold change in gene expression was determined using one way analysis of variance (ANOVA) across multiple groups, followed by unpaired two tailed *t* test to compare each successive time point with 72 h. A *p* value of ≤0.05 was considered statistically significant.

### Data availability statement

All data is available in its raw format and without reservations upon request.

## Electronic supplementary material

Supplementary Information
