# Supplementary material for: Isolation and characterisation of alveolar type II pneumocytes from adult bovine lung
Source: Sci Rep. 2018 Aug 9;8:11927. doi: 10.1038/s41598-018-30234-x (PMC6085293; doi:10.1038/s41598-018-30234-x)
Supplement: Supplementary file 1 — Supplementary Information [file 41598_2018_30234_MOESM1_ESM.docx]

**Scientific Reports**

**Supplementary Information**

**Isolation and characterisation of alveolar type II pneumocytes from adult bovine lung**

Diane Frances Lee, Francisco Javier Salguero, Duncan Grainger, Robert James Francis, Kirsty MacLellan-Gibson and Mark Andrew Chambers

**Supplementary Results**

**
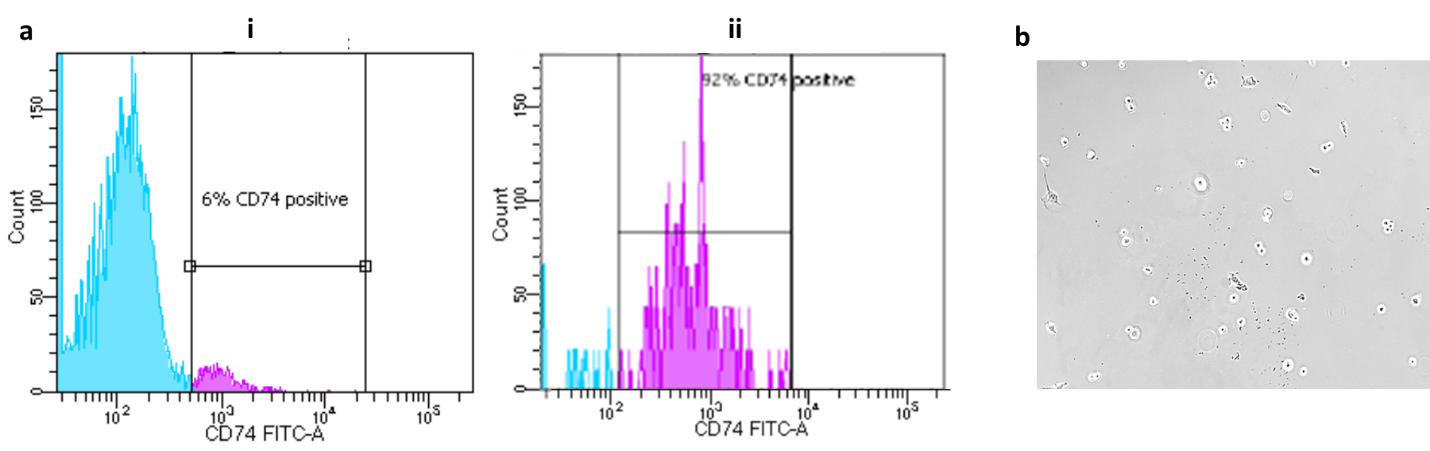
**

**Figure S1. ATII cells were isolated using FITC labelled CD74 as a marker for ATII.** (a) Of the sampled population, 6 % were found to be positive for anti-CD74 FITC, following digestion (i), whilst sorted cells were shown to be 92 % positive (ii). (b) Isolated cells, three days post-sorting, imaged using a Zeiss Axiovert 25 inverted microscope in brightfield mode, 20 x objective, showing mostly debris.

**
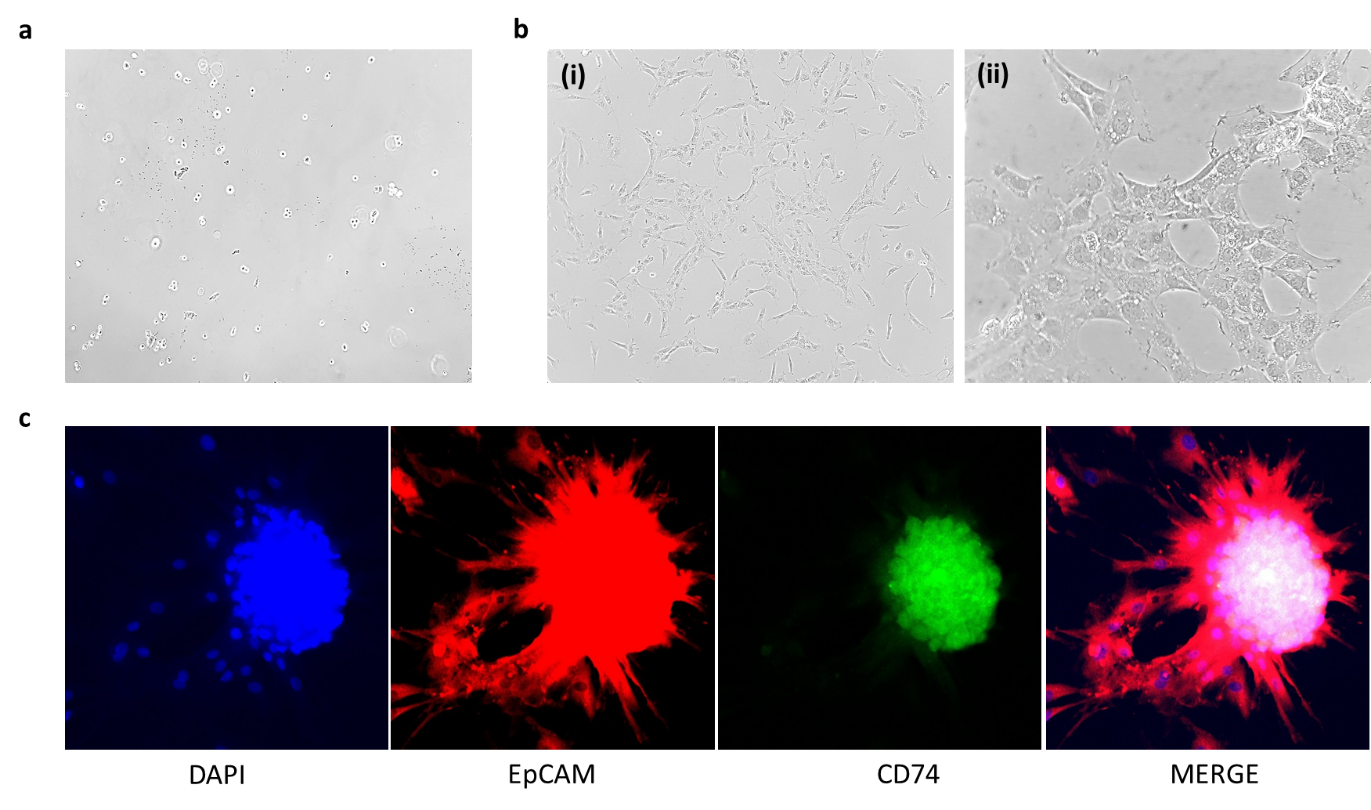
**

**Figure S2. Isolation of ATII cells using magnetic bead sorting.** (a) Debris, isolated by magnetic cell sorting of CD74-FITC positive cells from freshly digested tissue. (b) Isolated cells from a repeat of the magnetic sorting procedure performed on unsorted cells which were seeded directly in SAGM into T75 flasks. Images were acquired using a Zeiss Axiovert 25 inverted microscope in brightfield mode, 10 x objective (i) and 20 x objective (ii). (c) IF analysis of isolated cells grown on coverslips using methods outlined in main methods section. Images are representative of three replicates of the same isolation.

**Supplementary Experimental Procedures**

**FACS**

Distal lung tissue was processed from freshly slaughtered cattle as per the main methods section (isolation of alveolar type II (ATII) epithelial cells). Digested and sequentially filtered cells were incubated with anti-CD74 FITC clone 5-329 (Miltenyi Biotech, Germany) at a concentration of 1 µg/10^6^ cells for 20 min at 4°C in Dulbecco’s PBS/2 % FBS/1mM EDTA. Samples were sorted using a Becton Dickinson BD FACSAria Fusion and data analysed using BD FACSDiva Software v8.0.1 software (Becton Dickinson Immunocytometry Systems). All analyses were performed with an acquisition of 10,000 events. Anti-CD74 FITC positive cells were counted and seeded in small airway growth medium (PromoCell GmbH, Heidelberg, Germany).

**Magnetic Bead Sorting**

Distal lung tissue was processed from freshly slaughtered cattle as per the main methods section (isolation of alveolar type II (ATII) epithelial cells**).** Digested and sequentially filtered cells (1 x 10^7^ cells/100 μL in PBS pH 7.2, 0.5 % BSA and 2 mM EDTA) were incubated with anti-CD74 FITC clone 5-329 (Miltenyi Biotec, Bergisch Gladbach, Germany) according to manufacturer's recommendations for 10 minutes in the dark at 4−8 °C. Cells were washed twice in PBS pH 7.2, 0.5 % BSA and 2 mM EDTA to remove unbound antibody, before incubation with 10 μL anti-FITC MicroBeads (Miltenyi Biotec) per 10^7^ cells for 15 minutes in the dark at 4−8 °C. The wash steps were repeated and the suspension loaded onto an MS Column (Miltenyi Biotec). The column was placed in the magnetic field of an OctoMACS Separator (Miltenyi Biotec) and washed twice with PBS pH 7.2, 0.5 % BSA and 2 mM EDTA to remove unbound cells. The column was removed from the magnetic field and the recovered cells, positively selected for anti-CD74 FITC by the magnetic particle concentrator, re-suspended in SAGM and cultured on tissue culture treated 6-well plates. Cells negative for anti-CD74 FITC (‘unbound fraction’) were cultured in the SAGM until confluence. These cells were then subjected to a second round of anti-CD74 FITC magnetic cell sorting.

Anti-CD74 FITC positive cells isolated via the second magnetic sorting procedure were seeded either in T25 flasks or onto coverslips and analysed for markers of ATII cells by immunofluorescence, as outlined in the main methods section, using antibodies described therein.
